# A Universal Fast Algorithm for Sensitivity-Based Structural Damage Detection

**DOI:** 10.1155/2013/235820

**Published:** 2013-12-19

**Authors:** Q. W. Yang, J. K. Liu, C. H. Li, C. F. Liang

**Affiliations:** ^1^Department of Civil Engineering, Shaoxing University, Shaoxing 312000, China; ^2^Department of Mechanics, Sun Yat-Sen University, Guangzhou 510275, China

## Abstract

Structural damage detection using measured response data has emerged as a new research area in civil, mechanical, and aerospace engineering communities in recent years. In this paper, a universal fast algorithm is presented for sensitivity-based structural damage detection, which can quickly improve the calculation accuracy of the existing sensitivity-based technique without any high-order sensitivity analysis or multi-iterations. The key formula of the universal fast algorithm is derived from the stiffness and flexibility matrix spectral decomposition theory. With the introduction of the key formula, the proposed method is able to quickly achieve more accurate results than that obtained by the original sensitivity-based methods, regardless of whether the damage is small or large. Three examples are used to demonstrate the feasibility and superiority of the proposed method. It has been shown that the universal fast algorithm is simple to implement and quickly gains higher accuracy over the existing sensitivity-based damage detection methods.

## 1. Introduction

Recently, many methods have been presented to identify structural damage using the changes of modal parameters, specifically, the natural frequencies (or the square root of eigenvalues) and the mode shapes (i.e., the eigenvectors). In engineering practice, only a few eigenvalues and partial mode shapes can be obtained by a modal survey for large flexible structure. Then these existing damage identification methods can be categorized by solving the incomplete measurement problem. One group includes these methods in which mode shape expansion or model reduction cannot be avoided in damage identification. Many usual methods belong to this group, such as the finite element model (FEM), updated techniques based on the residual force vector [1–6], modal strain energy change methods [7, 8], and so forth. Methods of this sort will introduce additional errors into damage detection results, because the eigenvector expansion process would introduce errors in the “expanded” eigenvectors and the model reduction process would introduce errors in the FEM. The other group involves those methods that can detect structural damage by directly using the incomplete modal parameters without any eigenvector expansion or model reduction. The sensitivity-based techniques belong to this group, such as the eigenvalue sensitivity [9–12], the eigenvector sensitivity [13], the flexibility sensitivity [14–17], or the combined sensitivity [18–20]. These methods make use of the derivatives of modal parameters with respect to physical design variables. These sensitivity coefficients are then used to calculate changes in the parameters that would force the analysis frequencies and modes to match those measured in the test. Messina et al. proposed a damage detection method termed the multiple damage location assurance criterion (MDLAC) by using the eigenvalue sensitivity analysis [9]. Wong et al. developed an iterative method based on the general-order perturbation theory and optimization method for multiple structural damage detection [10]. Yu et al. make use of eigenvalue perturbation theory and artificial neural network to detect small structural damage [11]. Yang and Liu defined a damage localization criterion to locate structural damage firstly and then used the eigenvalue sensitivity method to obtain the damage extent [12]. Shi et al. used the eigenvector sensitivity analysis to determine the damage location firstly and then used the eigenvalue sensitivity method to obtain the damage extent [13]. Wu and Law studied the truncated modal flexibility sensitivity with the generic parameters in the system matrices [14]. This sensitivity has again been formulated and studied for the eigenparameters of the system matrices to detect structural damage [15]. By the matrix eigendecomposition and flexibility sensitivity analysis, Yang and Liu approache the damage identification problem in a decoupled fashion: determining the number of damaged elements, localizing the damaged elements, and quantifying the damage extents [16]. Li et al. proposed a generalized flexibility sensitivity method for structural damage detection [17]. Compared with the original flexibility matrix based approach, the effect of truncating higher-order modes can be considerably reduced in their method. Wong et al. proposed a perturbation method to detect damage of a multistorey building by combining the eigenvalue sensitivity with the eigenvector sensitivity [18]. Lam et al. presented a damage localization procedure based on the eigenvalue and eigenvector sensitivity analysis [19]. Using the Neumann series expansion, Yang derived the flexibility sensitivity and developed a mixed perturbation method to identify structural damage by combining the eigenvalue sensitivity with the flexibility sensitivity [20].

The sensitivity-based damage detection techniques generally require considerable computational expense for large damage case. It has been pointed out that when the change of structural parameter is more than 15%, the second order perturbation should be taken into account [21]. As an alternative, some researches [13] have used the iteration scheme to tackle the large damage case. It is anticipated that the computational cost of these existing sensitivity methods will be very expensive for large damage case, since a higher-order approximation should be performed or an iteration scheme must be used to estimate the damage extent more precisely. To combat this drawback, a universal fast algorithm is presented in this paper that can improve the calculation accuracy of the existing sensitivity-based technique without any high-order sensitivity analysis or multi-iterations, regardless of whether the damage is small or large. The key point of the fast algorithm lies in a simple accelerated formula, which is derived from the stiffness and flexibility matrix spectral decomposition theory in [22]. As will be shown in Section 4, with the introduction of the accelerated formula, the sensitivity-based method is able to accurately and quickly quantify structural damages without high-order sensitivity analysis or multi-iterations. The presentation of this work is organized as follows. In Section 2, the basis for the sensitivity-based damage detection methods is briefly reviewed. Then a fast algorithm is developed in Section 3 to quickly improve the calculation accuracy of the sensitivity-based technique. Moreover, the source of the accelerated formula is also discussed in detail in Section 3. Three examples are used in Section 4 to show the feasibility and the superiority of the proposed method. The conclusions of this work are summarized in Section 5. In the following theoretical development, it is assumed that structural damages only reduce the system stiffness matrix and structural refined FEM has been developed before damage occurrence.

## 2. Sensitivity-Based Methods 

In this section, the basis for the sensitivity-based damage detection methods is briefly reviewed. And then three existing sensitivity methods are introduced in detail, namely, the eigenvalue sensitivity, the flexibility sensitivity, and the generalized flexibility sensitivity.

Consider the analytical model of a given structure, with *n* degrees of freedom (DOFs), whose modes of vibration can be obtained by solving the following generalized eigenvalue problem:

(1) $$\begin{array}{c} K\phi_{j}=\lambda_{j}M\phi_{j}, \end{array}$$

where *M* and *K* are the mass and stiffness matrices and *λ*_*j*_ and *ϕ*_*j*_ are the *j*th eigenvalue and eigenvector, respectively. Since it is assumed that the damage can only cause change of stiffness, the global stiffness matrix *K* can be expressed as a function of elemental stiffness parameters, that is,

(2) K=∑i=1NKi=K({p}), ({p}={p1,p2,…,pN}T),

where *K*_*i*_ is the *i*th elemental stiffness matrix, {*p*} is the vector consisting of the elemental stiffness parameters *p*_*i*_ (*i* = 1 ~ *N*), and *N* is the total number of elements. Using Taylor or Neumann series expansion and keeping the first order item, we can obtain the following linear approximation expression:

(3) Sα=Δd, α={Δp1p1,Δp2p2,…,ΔpNpN}T,

where *S* is the sensitivity matrix representing the first order derivation of modal data {*d*} to {*p*}, Δ*d* is the change vector of modal data, *α* is the change of the stiffness parameters before and after damage, and *α*_*i*_ = Δ*p*_*i*_/*p*_*i*_ is the *i*th elemental stiffness perturbed parameter (i.e., the elemental damage parameter). The value of *α*_*i*_ is 0 if the *i*th element is undamaged and *α*_*i*_ is 1 or less than 1 if the corresponding element is completely or partially damaged. From (3), one has

(4) $$\begin{array}{c} \alpha =S^{+}\Delta d, \end{array}$$

where the superscript “+” denotes the generalized inverse. Equation (4) shows that the location and extent of damage (*α*) can be determined only if the changes of modal parameters Δ*d* are available through modal test. The main differences between the various sensitivity-based schemes are the test parameters Δ*d* used in (4) and the corresponding sensitivity matrix *S*. In the next section, three existing sensitivity methods are introduced, which will be used to demonstrate the merits of the proposed fast method.

### 2.1. The Eigenvalue Sensitivity Method

For the eigenvalue sensitivity analysis, the eigenvalues are measured in structural modal test and used in (4) to compute the stiffness perturbed parameters. The first order derivative of the *j*th eigenvalue can be computed by [9–12]

(5) $$\begin{array}{c} \frac{\partial \lambda_{j}}{\partial p_{i}}=\phi_{j}^{T}\frac{\partial K}{\partial p_{i}}\phi_{j}. \end{array}$$

Then, the eigenvalue sensitivity matrix *S*_1_ can be derived from (5) as

(6) $$\begin{array}{c} S_{1}=\left[\begin{array}{cccc} \frac{\partial \lambda_{1}}{\partial p_{1}} & \frac{\partial \lambda_{1}}{\partial p_{2}} & \cdots & \frac{\partial \lambda_{1}}{\partial p_{N}}\\ \frac{\partial \lambda_{2}}{\partial p_{1}} & \frac{\partial \lambda_{2}}{\partial p_{2}} & \cdots & \frac{\partial \lambda_{2}}{\partial p_{N}}\\ \vdots & \vdots & \ddots & \vdots \\ \frac{\partial \lambda_{m}}{\partial p_{1}} & \frac{\partial \lambda_{m}}{\partial p_{2}} & \cdots & \frac{\partial \lambda_{m}}{\partial p_{N}} \end{array}\right]. \end{array}$$

If *m* eigenvalues are available through modal test, *α*, that is, the structural damage, can be identified by (4).

### 2.2. The Flexibility Sensitivity Method

Using Neumann series expansion, the first-order sensitivity of structural flexibility matrix can be obtained as [14–17]

(7) $$\begin{array}{c} \frac{\partial F}{\partial p_{i}}=FK_{i}F, \end{array}$$

where *F* (*F* = *K* ^−1^) is the flexibility matrix of the intact structure. Then, the first-order sensitivity equation of structural flexibility matrix for all elemental damage parameters can be established as

(8) $$\begin{array}{c} \Delta F=\overset{N}{\underset{i=1}{\sum }}\alpha_{i}\frac{\partial F}{\partial p_{i}}. \end{array}$$

With mode shapes normalized to unit mass, the flexibility matrix change can be obtained approximately by a few low-frequency modes as

(9) $$\begin{array}{c} \Delta F=\overset{m}{\underset{j=1}{\sum }}\frac{1}{\lambda_{dj}}\phi_{dj}\phi_{dj}^{T}-\overset{m}{\underset{j=1}{\sum }}\frac{1}{\lambda_{j}}\phi_{j}\phi_{j}^{T}, \end{array}$$

where *λ*_*dj*_ and *ϕ*_*dj*_ are the *j*th eigenvalue and eigenvector of the damaged structure and *m* is the number of measured modes in modal survey. From (8) and (9), the unknown damage parameters *α*_*i*_ (*i* = 1 ~ *N*) can be readily computed by manipulating the matrix equation (8) into a set of linear equations.

### 2.3. The Generalized Flexibility Sensitivity Method

The generalized flexibility matrix *F* ^*g*^ for a structure with *n* degrees of freedom is defined as

(10) $$\begin{array}{c} F^{g}=FMF, \end{array}$$

where *F* and *M* are the (*n* × *n*) flexibility and mass matrices, respectively. The first order derivative of the generalized flexibility matrix can be computed by [17]

(11) $$\begin{array}{c} \frac{\partial F^{g}}{\partial p_{i}}=FK_{i}FMF+FMFK_{i}F. \end{array}$$

Then the sensitivity equation of the generalized flexibility matrix for all elemental damage parameters can be established as

(12) $$\begin{array}{c} \Delta F^{g}=\overset{N}{\underset{i=1}{\sum }}\alpha_{i}\frac{\partial F^{g}}{\partial p_{i}}, \end{array}$$

where Δ*F* ^*g*^ is the change of the generalized flexibility matrix. When damage is introduced, Δ*F* ^*g*^ can be approximately expressed by using only a few of the lower frequency modes as

(13) ΔFg=FdMFd−FMF≈∑j=1m1λdj2ϕdjϕdjT −∑j=1m1λj2ϕjϕjT,

where *F*_*d*_ is the damaged stiffness matrix. From (12) and (13), the unknown damage parameters *α*_*i*_ (*i* = 1 ~ *N*) can be computed by manipulating the matrix equation (12) into a set of linear equations.

## 3. The Universal Fast Algorithm 

As stated in Section 1, the above sensitivity techniques generally require considerable computational expense for large damage case, since a higher order approximation should be performed or an iteration scheme must be used to estimate the damage extent more precisely. In this section, a universal accelerated formula is developed to quickly improve the calculation accuracies of the above original sensitivity methods without any high-order sensitivity analysis or multi-iterations.

The fast algorithm consists of the following steps. (1) Compute the elemental damage parameters *α*_*i*_ (*i* = 1 ~ *N*) by any one of the existing sensitivity methods. (2) Use the universal accelerated formula to obtain the new value (*α*_*i*_^new^) of damage parameter for those elements with *α*_*i*_ ≥ 0.176. The accelerated formula is as follows:

(14) $$\begin{array}{c} \alpha_{i}^{\text{new}}=\frac{\alpha_{i}}{1+\alpha_{i}}. \end{array}$$

**Figure 1:**
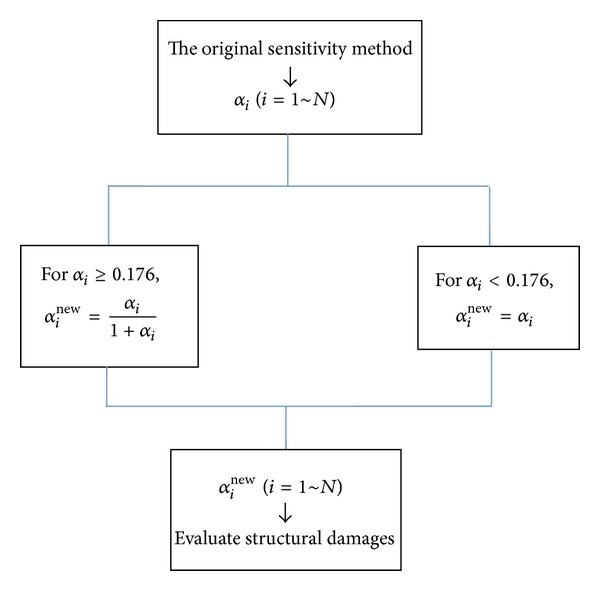
A graphical illustration of the universal fast algorithm.

Then the damage extent can be assessed again from the new result *α*_*i*_^new^ for those elements (i.e., *α*_*i*_^new^ = *α*_*i*_/(1 + *α*_*i*_) if *α*_*i*_ ≥ 0.176). (3) For those elements with *α*_*i*_ < 0.176, the original calculation result *α*_*i*_ will be as the final result *α*_*i*_^new^ (i.e., *α*_*i*_^new^ = *α*_*i*_ if *α*_*i*_ < 0.176). (4) In the end, structural damages can be evaluated by the resulting *α*_*i*_^new^ (*i* = 1 ~ *N*). The above steps are described in Figure 1. As will be shown in the examples in Section 4, with the introduction of the accelerated formula, structural damage extents can be quickly and accurately calculated without any high-order sensitivity analysis or multi-iterations, regardless of whether the damage is small or large.

The source of the accelerated formula (14) is illustrated as follows. In [22], the author proposed a new flexibility perturbation technique based on matrix spectral decomposition, which has a unique advantage that it can accurately compute the stiffness perturbation parameter without any higher-order sensitivity analysis or iteration. In this study, we will prove that this new flexibility perturbation method can be seen as a combination of the original flexibility sensitivity method and the above accelerated formula.

The new flexibility perturbation theory in [22] begins with the disassembly of the (*n* × *n*) global stiffness matrix, which can be obtained by the spectral decomposition of each elemental stiffness matrix [15, 16]. Generally, the elemental stiffness matrix *K*_*i*_ is not of full rank in most cases. Without loss of generality, for convenience of the following derivation, all the ranks of elemental stiffness matrices are presumed to be 1 (other cases with the rank greater than 1 are also valid). Using the spectral decomposition, the disassembly of the undamaged global stiffness matrix can be obtained as

(15) $$\begin{array}{c} K=\overset{N}{\underset{i=1}{\sum }}K_{i}=C\left[p\right]C^{T}, \end{array}$$

where

(16) $$\begin{array}{c} K_{i}=c_{i}p_{i}c_{i}^{T},\\ C=\left[c_{1},c_{2},\ldots c_{N}\right],\\ \left[p\right]=\left[\begin{array}{cccc} p_{1} & & & \\ & p_{2} & & \\ & & \ddots & \\ & & & p_{N} \end{array}\right]. \end{array}$$

The (*n* × *N*) matrix *C* is defined as the stiffness connectivity matrix representation of the connectivity between DOFs. According to (15), it is important to note that *N* ≥ *n* and the matrix *C* is of full rank (rank⁡(*C*_*n*×*N*_) = *n*), because *K* is of full rank (rank⁡(*K*_*n*×*n*_) = *n*). The matrix *C* is independent of [*p*] and unchanged as damage occurs. Then the global stiffness matrix of the damaged structure can be assembled as

(17) $$\begin{array}{c} K_{d}=\overset{N}{\underset{i=1}{\sum }}p_{i}\left(1-\alpha_{i}\right)K_{i}=C\left[p_{d}\right]C^{T}\\ \left[p_{d}\right]=\left[\begin{array}{cccc} p_{1}\left(1-\alpha_{1}\right) & & & \\ & p_{2}\left(1-\alpha_{2}\right) & & \\ & & \ddots & \\ & & & p_{N}\left(1-\alpha_{N}\right) \end{array}\right]. \end{array}$$

For the case of *n* = *N*, the disassemblies of the (*n* × *n*) global flexibility matrices *F* and *F*_*d*_, for the undamaged and damaged structure, can be obtained by *F* · *K* = *F*_*d*_ · *K*_*d*_ = *I*_*n*×*n*_ as

(18) $$\begin{array}{c} F=K^{-1}={\left(C^{T}\right)}^{-1}{\left[p\right]}^{-1}C^{-1} \end{array}$$

(19) $$\begin{array}{c} F_{d}=K_{d}^{-1}={\left(C^{T}\right)}^{-1}{\left[p_{d}\right]}^{-1}C^{-1}. \end{array}$$

Subtracting (18) from (19), the flexibility matrix perturbation Δ*F* can be given as

(20) $$\begin{array}{c} \Delta F={\left(C^{T}\right)}^{-1}\left[{\left[p_{d}\right]}^{-1}-{\left[p\right]}^{-1}\right]C^{-1}. \end{array}$$

Equation (20) can be rewritten as

(21) $$\begin{array}{c} \Delta F={\left(C^{T}\right)}^{-1}{\left[p\right]}^{-1}\left[\beta \right]C^{-1}, \end{array}$$

where

(22) $$\begin{array}{c} \left[\beta \right]=\left[\begin{array}{cccc} \beta_{1} & & & \\ & \beta_{2} & & \\ & & \ddots & \\ & & & \beta_{N} \end{array}\right] \end{array}$$

(23) $$\begin{array}{c} \beta_{i}=\frac{\alpha_{i}}{1-\alpha_{i}}, \end{array}$$

where *β*_*i*_ is defined as the *i*th elemental flexibility perturbed parameter. According to the matrix theory, we have

(24) $$\begin{array}{c} {\left(C^{T}\right)}^{-1}{\left[p\right]}^{-1}={\left(C^{T}\right)}^{-1}\cdot {\left[p\right]}^{-1}\cdot \left(C^{-1}C\right)=FC \end{array}$$

(25) C−1=[p]·[p]−1C−1=[p](CT·(CT)−1)[p]−1C−1=[p]CTF.

Substituting (22), (24), and (25) into (21), one has

(26) ΔF=FC[β][p]CTF=∑i=1NβiF(cipiciT)F=∑i=1NβiFKiF.

Substituting (7) into (26) yields

(27) $$\begin{array}{c} \Delta F=\overset{N}{\underset{i=1}{\sum }}\beta_{i}\frac{\partial F}{\partial p_{i}}. \end{array}$$

The implication of (27) is very important. Compared with (8), (27) shows that the results obtained by (8) are not the stiffness perturbed parameters in a real sense, but the flexibility perturbed parameters. And the true stiffness perturbed parameter should be computed by using (23) as

(28) $$\begin{array}{c} \alpha_{i}=\frac{\beta_{i}}{1+\beta_{i}}. \end{array}$$

In view of the traditional sensitivity technique, (28) can be seen as an accelerated operation based on the results obtained by the original sensitivity method. Therefore, the accelerated formula (14) can be obtained by replacing *β*_*i*_(*α*_*i*_) with *α*_*i*_(*α*_*i*_^new^) in (28). For the case of *n* = *N*, we can use the generalized inverse “+” instead of the inverse “−” in the above derivation and the same accelerated formula as (14) can be obtained. In addition, the critical value 0.15 in the traditional sensitivity method also changes to be 0.176 (according to (23), the new critical value is 0.15/(1 − 0.15) = 0.176).

## 4. Numerical Examples

To illustrate the feasibility and superiority of the universal fast algorithm, three numerical examples are presented to show the improvement of the existing sensitivity methods by using the universal fast algorithm.

**Example 1**

**Figure 2:**
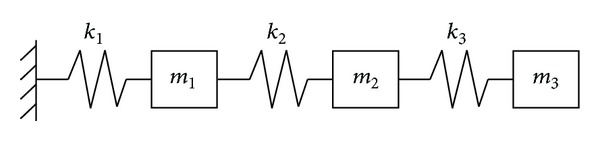
Spring-mass system (Example 1).

The first example is a spring-mass system with 3 DOFs as shown in Figure 2, which is used to compare the performance of the universal fast algorithm and the original flexibility sensitivity method. Consider the nominal model of the system to have the parameters *k*_*i*_ = 1 (*i* = 1 ~ 3) and *m*_*j*_ = 1 (*j* = 1 ~ 3). Three damage cases are studied in the example. Case 1: element 2 is damaged with *k*_2_ = 0.85. Case 2: element 2 is damaged with *k*_2_ = 0.2. Case 3: elements 2 and 3 are damaged with *k*_2_ = 0.4 and *k*_3_ = 0.5.  Table 1 presented the results obtained by the original flexibility sensitivity method and the proposed fast algorithm. In Table 1, the results of the fast algorithm are calculated by using (14). For example, in Table 1, *α*_2_^new^ = *α*_2_/(1 + *α*_2_) = 0.1765/(1 + 0.1765) = 0.15 for damage case 1, *α*_2_^new^ = *α*_2_/(1 + *α*_2_) = 4/(1 + 4) = 0.8 for damage case 2, and so on.  Table 1 shows that the stiffness perturbated parameters (i.e., the damage parameters) can be exactly computed for this example by the universal fast algorithm if the complete and exact modes are given, regardless of whether the damage is small or large.

**Example 2**

**Figure 3:**
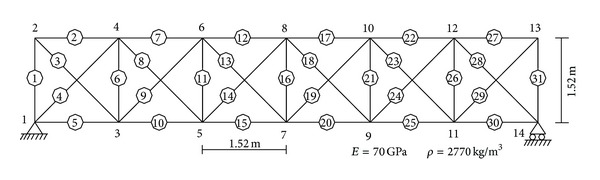
Thirty-one-bar truss structure (Example 2).

The second example is the two-dimensional truss structure (shown in Figure 3) used by Shi et al. [13], which is employed to compare the performance of the universal fast algorithm and the iterative eigenvalue sensitivity method used in [13]. Three damage cases (listed in Table 2) are studied in [13].  Table 3 lists the values of damage parameters calculated by the original iterative sensitivity method in [13]. From Table 3, it is obvious that results obtained in the first iteration all have large errors compared to the true values, regardless of whether the noise is considered or not. Although more accurate results can be obtained with the iteration number increasing, the computational cost of this iterative sensitivity method is very expensive, because the sensitivity matrix must be recalculated in each iteration.  Table 4 lists the results obtained by the proposed fast algorithm. The values in Table 4 are achieved by using the accelerated formula (14) on the basis of the values of the first iteration in Table 3. For example, the value 0.286 in Table 4 is obtained by *α*_16_^new^ = *α*_16_/(1 + *α*_16_) = 0.4/(1 + 0.4) = 0.286, the value 0.292 in Table 4 is obtained by *α*_1_^new^ = *α*_1_/(1 + *α*_1_) = 0.412/(1 + 0.412) = 0.292, and so on. The values 0.134, 0.172, and 0.154 in Table 3 are directly used as the final results in Table 4, because they are all less than 0.176. From Table 4, one can see that the result obtained by the proposed method is more accurate than the results in Table 3 achieved by the iterative sensitivity method after three iterations. It can be seen from Tables 3 and 4 that the results of the presented method have equivalent accuracy to that of the iterative scheme after two or three iterations. It has been shown that the proposed method can achieve satisfactory results without any higher-order approximation or multi-iterations.

**Example 3**

**Figure 4:**
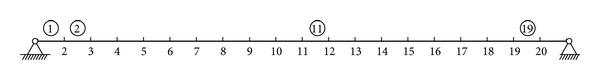
A simple supported beam (Example 3).

The third example is a simple supported beam (shown in Figure 4) used by Li et al. in [17], which is used to compare the performance of the universal fast algorithm and the generalized flexibility sensitivity method. In [17], multiple damages are simulated in elements 2, 11, and 19 with stiffness losses of 15%, 20%, and 10%, respectively. Results computed by the original generalized flexibility sensitivity method in [17] are listed in Table 5. Using the accelerated formula, results obtained by the universal fast algorithm are also listed in Table 5 for comparison. As before, the new damage parameter *α*_*i*_^new^ is calculated by (14). For example, in Table 5, *α*_2_^new^ = *α*_2_/(1 + *α*_2_) = 0.1777/(1 + 0.1777) = 0.1509. It can be concluded from Table 5 that the universal fast algorithm can achieve more accurate results than that obtained by the original generalized flexibility sensitivity method.

## 5. Conclusions

A universal fast algorithm for sensitivity-based structural damage detection has been developed in this study, which can improve the calculation accuracy of the sensitivity-based technique without any high-order sensitivity analysis or multi-iterations, regardless of whether the damage is small or large. The key point of the fast algorithm lies in a simple accelerated formula, which is derived from the stiffness and flexibility matrix spectral decomposition theory. Three examples are used to exercise this process and measurement noise is also simulated in damage detection. The results show the superiority of the proposed method over the original sensitivity-based methods in the identification of structural damages. It has been shown that the proposed procedure may be a promising method in structural damage detection.

## Figures and Tables

**Table 1:** Comparisons of the estimated damage parameters by the original flexibility sensitivity method and the fast algorithm (Example 1).

Damage case	Element no.	True damage reduction	Damage parameters computed by the original flexibility sensitivity method	Damage parameters computed by the universal fast algorithm
1	2	0.15	0.1765	0.15
2	2	0.8	4	0.8
3	2, 3	0.6, 0.5	1.5, 1	0.6, 0.5

**Table 2:** Damage scenarios studied in [13] (Example 2).

Case 1		Case 2		Case 3	
Element no.	Damage	Element no.	Damage	Element no.	Damage
16	30%	1	30%	7	15%
		2	20%	11	20%
				12	15%

**Table 3:** Results of the calculated damage parameters in [13] (Example 2).

Damage case	Element no.	True damage reduction	No noise		With noise		
			Iteration 1	Iteration 2	Iteration 1	Iteration 2	Iteration 3
1	16	0.3	**0.400** (**33.3%**)	0.311 (3.7%)	**0.482** (**60.7%**)	0.421 (40.3%)	0.378 (26%)
2	1	0.3	**0.412** (**37.3%**)	0.317 (5.7%)	**0.452** (**50.7%**)	0.342 (14%)	0.318 (6%)
	2	0.2	**0.260** (**30%**)	0.207 (3.5%)	**0.312** (**56%**)	0.248 (24%)	0.241 (20.5%)
3	7	0.15	**0.18** (**20%**)	0.149 (0.7%)	**0.182** (**21.3%**)	0.141 (6%)	0.140 (6.7%)
	11	0.2	**0.236** (**18%**)	0.205 (2.5%)	**0.172** (**14%**)	0.194 (3%)	0.196 (2%)
	12	0.15	**0.134** (**10.7%**)	0.149 (0.7%)	**0.144** (**4%**)	0.149 (0.7%)	0.147 (2%)

*The value in bracket denotes the comparative error between the calculated value and the true value.

**Table 4:** The results obtained by the universal fast algorithm (Example 2).

Damage case	Element no.	True damage reduction	No noise	With noise
1	16	0.3	0.286 (4.7%)↓	0.325 (8.3%)↓
2	1	0.3	0.292 (2.7%)↓	0.311 (3.7%)↓
	2	0.2	0.206 (3%)↓	0.238 (19%)↓
3	7	0.15	0.153 (2%)↓	0.154 (2.7%)↓
	11	0.2	0.191 (4.5%)↓	**0.172** (**14%**)
	12	0.15	**0.134** (**10.7%**)	**0.144** (**4%**)

*The value in bracket denotes the comparative error between the calculated value and the true value. “↓” denotes the decrease in error.

**Table 5:** Comparisons of the estimated damage parameters by the original generalized flexibility sensitivity method and the fast algorithm (Example 3).

Element no.	True damage reduction	Damage parameters obtained in [17]	Damage parameters computed by the universal fast algorithm
2	0.15	0.1777 (18%)	0.1509 (0.6%)↓
11	0.2	0.2564 (28.2%)	0.2041 (2.1%)↓
19	0.1	0.1120 (12%)	0.1120 (12%)

*The value in bracket denotes the comparative error between the calculated value and the true value. “↓” denotes the decrease in error.
